# SNPs selection using support vector regression and genetic algorithms in GWAS

**DOI:** 10.1186/1471-2164-15-S7-S4

**Published:** 2014-10-27

**Authors:** Fabrízzio Condé de Oliveira, Carlos Cristiano Hasenclever Borges, Fernanda Nascimento Almeida, Fabyano Fonseca e Silva, Rui da Silva Verneque, Marcos Vinicius GB da Silva, Wagner Arbex

**Affiliations:** 1 Federal University of Juiz de Fora - UFJF, Juiz de Fora, Minas Gerais, Brasil; 2 State of Minas Gerais Research Support Agency - FAPEMIG, Brasil; 3 Federal University of Viçosa - UFV, Viçosa, Minas Gerais, Brasil; 4 Brazilian Agricultural Research Corporation - Embrapa, Juiz de Fora, Minas Gerais, Brasil

**Keywords:** Single nucleotide polymorphisms, GWAS, support vector regression, wrapper, genetic algorithms, Pearson Universal kernel

## Abstract

**Introduction:**

This paper proposes a new methodology to simultaneously select the most relevant SNPs markers for the characterization of any measurable phenotype described by a continuous variable using Support Vector Regression with Pearson Universal kernel as fitness function of a binary genetic algorithm. The proposed methodology is multi-attribute towards considering several markers simultaneously to explain the phenotype and is based jointly on statistical tools, machine learning and computational intelligence.

**Results:**

The suggested method has shown potential in the simulated database 1, with additive effects only, and real database. In this simulated database, with a total of 1,000 markers, and 7 with major effect on the phenotype and the other 993 SNPs representing the noise, the method identified 21 markers. Of this total, 5 are relevant SNPs between the 7 but 16 are false positives. In real database, initially with 50,752 SNPs, we have reduced to 3,073 markers, increasing the accuracy of the model. In the simulated database 2, with additive effects and interactions (epistasis), the proposed method matched to the methodology most commonly used in GWAS.

**Conclusions:**

The method suggested in this paper demonstrates the effectiveness in explaining the real phenotype (PTA for milk), because with the application of the wrapper based on genetic algorithm and Support Vector Regression with Pearson Universal, many redundant markers were eliminated, increasing the prediction and accuracy of the model on the real database without quality control filters. The PUK demonstrated that it can replicate the performance of linear and RBF kernels.

## Background

Single nucleotide polymorphisms (SNPs) are an abundant form of genomic variation, which differ from rare variants [1] and the basic assumption for wide association studies (GWAS) is that the evaluated characteristic can be explained from this type of marker. Thus, it is considered that there are SNPs in the genotype with high Linkage Disequilibrium (LD) compared to Quantitative Trait Locus (QTL). So, the traditional approach is to evaluate which markers that have a high association with the phenotype through the p-value of beta linear regression between each SNP and the phenotype. After this step, the most relevant SNPs are analyzed for proximity to some region that is associated with that feature or other features that can be indirectly correlated with the phenotype in question. So far, the prediction of disease risk in humans based on validated SNPs based on this methodology showed little predictive power [2], although these SNPs indicate highly significant association with the phenotypic trait. This fact can be explained due the variance of the most significant markers have low explanatory power in relation to the phenotypic variance [3]. Therefore, an alternative approach is to increase the number of markers, considering also those with small correlations on the trait. But, this fact creates two problems: the number of markers is high and many of them are correlated. According to [4], such analysis requires the use of statistical methods that consider the selection of covariates (problem of multicollinearity) and the regularization of the estimation process (problem of dimensionality). Other regression techniques were created to address this problem as ridge regression and partial least squares regression [5]. On the other hand, machine learning algorithms such as Support Vector Machine (SVM) in GWAS considering multiple markers in classification problems, have demonstrated satisfactory performance as in [2], [6] and [7].

This study aims to propose a method that can simultaneously evaluate several SNPs in relation to the phenotype described by a continuous variable, unlike case-control dichotomous phenotypes addressed to the majority of GWAS studies. With this, there are three immediate benefits relative to standard methodology: one relating to the various levels of the phenotypes, the other by complex simultaneous interactions that may occur between the various markers and, finally, the use of evolutionary computation to properly select the main SNPs. Recently, some studies are being conducted with continuous phenotypes and Support Vector Regression (SVR) as in [8], [9] and [10]. However, the methodology used in these studies did not make use of metaheuristics to optimize the selection of markers as is done in this work.

With the evolution of new chips for cattle, with densities from 500,000 to 800,000 markers and if the genetic structure is defined by an underlying large number of small QTL, the formation of large data sets for training and precise phenotypic measures are needed to perform the improvement of the accuracy in increasing the density of SNPs [11]. Consequently, it is extremely important that new methodologies are developed to deal adequately with high-dimensional genomic data without the elimination of relevant variables. Therefore, after identifying the subset of sufficient and necessary markers for the explanation of the phenotype, it is possible to reduce costs in the manufacturing of custom chips with fewer SNPs to predict phenotype from genomic selection methods.

## Method

In this section, the two main techniques, SVR and GA (Genetic Algorithm), used in the construction of the suggested method will be discussed to demonstrate their potential advantages.

### Support vector regression

The first version SVM for regression was proposed in 1996 by Vladimir N. Vapnik, Harris Drucker, Christopher J. C. Burges, Linda Kaufman and Alexander J. Smola [12]. This technique is called SVR.

Let the set $\left\{\left(x_{1},y_{1}\right),\left(x_{2},y_{2}\right),...,\left(x_{n},y_{n}\right)\right\}$ with $x_{i}\in {\mathbb{R}}^{d}$and$y_{i}\in \mathbb{R}$. The goal of SVR is to find the linear functional *f*, described by Equation 1, which maps variables of the input space in the variable output space, minimizing the Expression 2.

(1) $$f\left(x\right)=\left\langle w,x\right\rangle +b\;\text{with}\;w,x\in {\mathbb{R}}^{d}$$

where $\left\langle w,x\right\rangle =w_{1}x_{1}+w_{2}x_{2}+...+w_{d}x_{d}$ is inner product between vectors $w,x\in {\mathbb{R}}^{d}$.

Let *w *and *b*, respectively, the slope and intercept of the hyperplane to be estimated from Expression 2.

(2) $$\underset{w,b}{\text{Minimize}}\;\frac{1}{2}{∥w∥}^{2}+C\overset{n}{\underset{i=1}{\sum }}L_{\varepsilon }\left(f\left(x_{i}\right),y_{i}\right)$$

where $∥w∥=\sqrt{w_{1}^{2}+w_{2}^{2}+...+w_{d}^{2}}$ is the *L_2 _*norm of the vector $w\in {\mathbb{R}}^{d}$.

where,

(3) $$L_{\varepsilon }\left(f\left(x_{i}\right),y_{i}\right)=\left\{\begin{array}{c} \begin{array}{ccc} 0 & if & \left|y_{i}-f\left(x_{i}\right)\right|\leq \varepsilon \end{array}\\ \begin{array}{ccc} \left|y_{i}-f\left(x_{i}\right)\right| & if & \left|y_{i}-f\left(x_{i}\right)\right|>\varepsilon \end{array}. \end{array}\right)$$

According to Expression 2, the term $\frac{1}{2}{∥w∥}^{2}$ indicates the complexity of the model and the term $L_{\varepsilon }\left(f\left(x_{i}\right),y_{i}\right)$ reflects the loss function ε-insensitive that does not penalize the values inside the tube, or smaller than ε as shown in Equation 3. The parameter *C *is called the regularization constant and reflects the balance between complexity *f *and the amount of greater deviations that will be tolerated [16]. Thus, the lower the tube (lower ε), the more complex the function *f *and, contrary, the larger the tube (greater ε) less complexity is required to *f*. The parameters *C *and ε need to be optimized to find a model suitable to the data [13].

With the introduction of appropriate slack variables $\xi_{i}$, $\xi_{i}^{*}$ and necessary algebraic manipulations, Expression 5 becomes the objective function of the Expression 4. Such a formulation is called the primal because the regression is based on the original data space [13]. The slack variables are intended to enable the occurrence of vector outside of the tube, and they are called support vectors, since they are the only ones contributing to the decline (Expression 5). Thus, all the other vectors in the tube may be removed after construction of the model [13]. This property allows the SVR to model relations in which the number of dependent variables is much larger than the size of the sample.

(4) $$\underset{w,b,\xi ,\xi^{*}}{\text{Minimize}}\frac{1}{2}{∥w∥}^{2}+C\overset{n}{\underset{i=1}{\sum }}\left(\xi_{i}+\xi_{i}^{*}\right)$$

Subject to:

(5) $$\left\{\begin{array}{c} y_{i}-\left\langle w,x_{i}\right\rangle -b\leq \varepsilon +\xi_{i}\\ \left\langle w,x_{i}\right\rangle +b-y_{i}\leq \varepsilon +\xi_{i}^{*}\\ \varepsilon ,\xi_{i},\xi_{i}^{*}\geq 0 \end{array}\right)$$

If the space of the original data doesn't have a linear relationship with the dependent variable, the function *f *is reformulated from the primal model to the dual model Expression 6). With this, the original space is mapped to a new space, called feature space by means of the function *ϕ* and the inner product $K\left(x_{i},x_{j}\right)=\left\langle \phi \left(x_{i}\right),\phi \left(x_{j}\right)\right\rangle$, where *K *is called the kernel function. This function reflects the underlying relationship between the input and output [13].

(6) $$f\left(x\right)=\left[\overset{n}{\underset{i=1}{\sum }}\left(\alpha_{i}-\alpha_{i}^{*}\right)\left\langle \phi \left(x_{i}\right),\phi \left(x\right)\right\rangle \right]+b$$

The dual variables $\alpha_{i}$ and $\alpha_{i}^{*}$ represents the Lagrange multipliers which satisfy the inequalities $0\leq \alpha_{i},\alpha_{i}^{*}\leq C$ which can be obtained by Expression 7 and by Equation 8.

(7) $$\underset{\alpha ,\alpha^{*}}{\text{Maximize}}\left\{\begin{array}{c} -\frac{1}{2}\overset{n}{\underset{i,j=1}{\sum }}\left(\alpha_{i}-\alpha_{i}^{*}\right)\left(\alpha_{j}-\alpha_{j}^{*}\right)K\left(x_{i},x_{j}\right)\\ -\varepsilon \overset{n}{\underset{i=1}{\sum }}\left(\alpha_{i}-\alpha_{i}^{*}\right)+\overset{n}{\underset{i=1}{\sum }}y_{i}\left(\alpha_{i}-\alpha_{i}^{*}\right) \end{array}\right)$$

Subject to:

(8) $$\left\{\begin{array}{c} \overset{n}{\underset{i=1}{\sum }}\left(\alpha_{i}-\alpha_{i}^{*}\right)=0\\ 0\leq \alpha_{i},\alpha_{i}^{*}\leq C \end{array}\right)$$

The main advantage of using the kernel function is a linear mapping between the input data transformed by the function ϕ and output data *y_i_*. This is possible because the feature space has a dimension greater than the dimension of the original space. Thus, the linear regression is obtained in the feature space rather than in the original space. Thus, the linear kernel is given by Equation 9, where it is observed that this kernel has no specific parameter.

(9) $$K\left(x_{i},x_{j}\right)=\left\langle x_{i},x_{j}\right\rangle$$

The kernel radial basis function (RBF) is a kernel of a general purpose when there is no a priori knowledge about the data [14]. This kernel given by Equation 10 has the parameter γ that must be chosen appropriately.

(10) $$K\left(x_{i},x_{j}\right)=\text{exp}\left(-\gamma {∥x_{i}-x_{j}∥}^{2}\right)$$

Pearson Universal Kernel VII (PUK), given by the Expression 11, was adopted as the kernel for the methodology proposed in this article. A Pearson VII function is able to easily change and adapt its two parameters σ and ω between the shapes of the Lorentzian and Gaussian function and even other functions [13]. Thus, this kernel has robustness like showing that percentage changes in parameters cause significant variations in the lower percentage in the RMSE of the predictions [13]. So, the PUK can replace the commonly applied kernels (Linear, Polynomial and RBF), possibly resulting in equal or superior performance regarding to the generalization of SVR [13]. The main advantage of this methodology is the choice of replacing the stock kernels by choosing the best parameters of kernel PUK. This exchange of choice creates a clear gain, as each kernel is adopted in the training necessary to optimize its parameters and in the case of PUK, the kernel is not exchanged for another, because it mimics the behavior of other kernels.

(11) $$K\left(x_{i},x_{j}\right)=\frac{1}{{\left[1+\left(\frac{2\sqrt{{∥x_{i}-x_{j}∥}^{2}}\sqrt{2^{\left(1/\omega \right)}-1}}{\sigma }\right)\right]}^{\omega }}$$

When checking the similarity of results for the kernels evaluated by [13], the linear and RBF kernels were also computed. The intention was to verify that, with the appropriate parameters, the PUK has performance equal or superior to other kernels.

### Genetic algorithms

Genetic algorithms are search algorithms based on the genetic processes and natural selection [15]. It simulates the evolution through three genetic operators: selection, crossover and mutation. An initial population is generated with a given size, then, is held to select individuals who will participate in the reproduction process. After the selection, it is made the crossover (or recombination) and, successively, mutation is applied to individuals. The operators of crossover and mutation are based on probabilities, so not all individuals will make crossover and/or mutation. The criterion for the parade can be the number of generations or a threshold error rate established. Genetic algorithms can be used for variable selection both in classification problems as for regression problems. Its great advantage is that it performs a smart search on some candidates, ensuring a constant improvement of individuals, but simultaneously inserting dispersion in the current population. The encoding of each individual must be binary and its fitness is the MSE (mean square error) of the selected subset of the original group variables, which are coded with 1, and the variables that are not selected are coded 0. Thus, you can enter the MSE of a specific kernel SVR as fitness GA to select a "good" subset of SNPs when the sample size is much smaller than the number of markers. The wrapper of this framework is described in detail in [16].

### Proposed method

The steps of the suggested method are:

1) The p-value of the Spearman's correlation of each marker with the phenotype is calculated.

2) To group the markers through the p-value in order to build an increasing sequence of subsets of markers.

3) Turn the SVR for the groups constructed in step 2 with kernel PUK, whose parameters are optimized, and choose the group with the best performance, that is, we make the first selection of markers.

4) From the best group reported in the previous step, we use the Genetic Algorithm (GA) to perform a second selection marker.

The goal of step 1 is to consider all markers with significant effects on the phenotype.

The step 2 aims to separate the markers into increasingly larger groups, easing the entry of less significant markers.

The step 3 evaluates the performance of the group of SNPs in relation to SVR PUK, in order to capture interactions among the SNPs that were not mapped in step 1. Thus, when two SNPs with small main effects are analyzed together they can generate more interaction effect than when analyzed separately. The same can occur with two SNPs with contrary magnitudes, one with high and one with low main effect.

**Figure 1:**
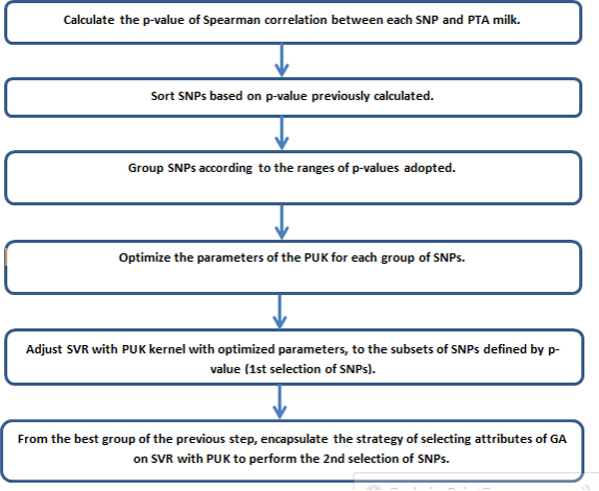
**Flowchart description of the proposed method**.

The step 4 aims to reduce the number of redundant markers that are highly correlated with the main markers associated with QTLs. This is because in the previous step no such filtering is performed. In addition, the complete search space of all combinations of markers is 2*^n ^*(where *n *is the number of SNPs), that is extremely prohibitive. Therefore, the GA will select a sample of different combinations of markers to evolve it toward a "good" combination by genetic operators called crossover and mutation. The general idea of the method is demonstrated in the flowchart in Figure 1.

## Material

To demonstrate the potential of the proposed methodology, two databases, one with only main effects without interactions between the markers, and another, with epistasis, which was generated by the function *simulateSNPglm *of the *scrime *package of the R software. No filters HWE and *call rate *in simulated databases were used, since there are no missing values and no markers in Hardy-Weinberg disequilibrium.

In order to check the method in a scenario with real noises, the method was tested on a real basis composed of genotype of bulls genotyped from Gyr provided by the Brazilian Agricultural Research Corporation (Embrapa), and only 244 animals have female offspring, allowing the measurement of the phenotype evaluated.

The suggested method was applied with and without Bonferroni [17] correction in both simulated databases, aiming to show that this "protection" can eliminate relevant markers for the phenotype, and to justify the no application of such correction in the p-value of the markers the real database.

### Genotype

The bovine genome has approximately 3 billion pairs of bases. Besides, it has 30 pairs of chromosomes, 29 autosomal pairs and 1 sexual pair. The genotype was 8 generated from the Illumina 56K chip, having a total of 56,947 markers. So, the explanatory variables, described by the frequency of allele B in locus, were coded as follows: AA = 0 (absence of the allele B), AB = 1 (presence of one copy of allele B) and BB = 2 (presence of two copies of allele B). Missing values due to reading errors, were considered as heterozygous AB = 1. In both simulated databases, the genotype is encoded as follows: 1 codes for the homozygous reference genotype, 2 for the heterozygous genotype, and 3 for the homozygous variant genotype, and a minus before these numbers means that the corresponding SNP should be not of this genotype. Both simulated phenotypes are also continuous variables.

### Real phenotype

The genetic potential of milk of an animal is computed from the milk production of their female offspring based on the methodology developed in [18]. The PTA milk is the predicted transmitting ability (PTA), being a measure of the expected performance of the daughters of the bull in relation to the average genetic herds. Therefore, for example, a PTA equal to 500 for milk production means that if the bull is used in a population with genetic level same as to that used to evaluate it, each daughter will produce an average of 500 kg per lactation more than the average herd. Considering two bulls, a with 500 kg PTA and other with -100 kg, it is expected that, in random mating, the daughters of the first bull will produce an average of 600 kg more than the daughters of the second bull.

The main difference from the studies presented in [2] and [6] on the selection of markers, is the exchange from classification problems (SVM) to regression problems (SVR) as the PTA milk is mapped by a continuous variable. This enables us to differentiate various levels of phenotypic trait, unlike problems of case-control classification.

For the calculation of PTA milk, only the genetic effect is considered, eliminating all other environmental effects. Like this, the explanation of PTA from molecular marker information is consistent. According to Table 1 it was possible to notice a wide range of values of PTA, indicating the need of robust models for this mapping measurement through the genotype.

**Table 1:** Statistics of the PTA for milk and of the simulations 1 and 2.

Database	Minimum	1º Quartile	Median	Mean	3º Quartile	Maximum
Real	-479.5	328.0	583.2	641.3	908.3	1,978.0
Simulation 1	-12.6	200.9	396.8	378.0	594.8	1,296.0
Simulation 2	-3.163	-0.2049	1.4320	57.66	149.4	301.3

### Simulated phenotype without epistasis (simulation 1)

The generated model is described by Equation (12).

(12) $$Y=\beta_{0}+\beta_{1}L_{1}+\beta_{2}L_{2}+\beta_{3}L_{3}+\beta_{4}L_{4}+\beta_{5}L_{5}+\beta_{6}L_{6}+\beta_{7}L_{7}+error$$

Where *error *is a normal random variable with mean 0 and standard deviation 5, L1 = (SNP1 == 2), L2 = (SNP10 == 1), L3 = (SNP20 == 3), L4 = (SNP30 == 3), L5 = (SNP40 == 3), L6 = (SNP50 == 2), L7 = (SNP60 == 2) and *Y *is simulated phenotype. The beta coefficients were set as $\beta_{0}=0$, $\beta_{1}=200$, $\beta_{2}=200$, $\beta_{3}=200$, $\beta_{4}=900$, $\beta_{5}=\beta_{6}=\beta_{7}=200$. A thousand markers were simulated for 250 subjects, with a minor allelic frequency (MAF), simulated for each SNP, based on a uniform distribution with minimum and maximum limits, respectively, 0.10 and 0.40. The linkage disequilibrium (LD) is calculated indirectly by MAF, ie, no there is an input parameter to control this variable. The variable *Y *of Equation (12) is continuous. The simulated databases 1 and 2 formed by the genotype-phenotype can be reconstructed from the sup file 1.

### Simulated phenotype with epistasis (simulation 2)

The generated model is described by the Equation (13).

(13) $$Y=\beta_{0}+\beta_{1}L_{1}+\beta_{2}L_{2}+\beta_{3}L_{3}+error$$

Where *error *is a normal random variable with mean 0 and standard deviation 1, L1 = (SNP4 != 2)^3 ^& (SNP3 != 1), L2 = (SNP5 == 3), L3 = (SNP12 != 1) & (SNP9 == 3) and Y is simulated phenotype. The beta coefficients were set as$\beta_{0}=0$, $\beta_{1}=\beta_{2}=150$ e $\beta_{3}=40$. Ten thousand markers were simulated for 600 subjects, MAF, simulated for each SNP, based on a uniform distribution with minimum and maximum limits as used on the simulated phenotype without epistasis (sup file 1).

^3^The SNP1 != 2 symbol means that the SNP1 does not have the largest allele frequency represented by 2.

**Figure 2:**
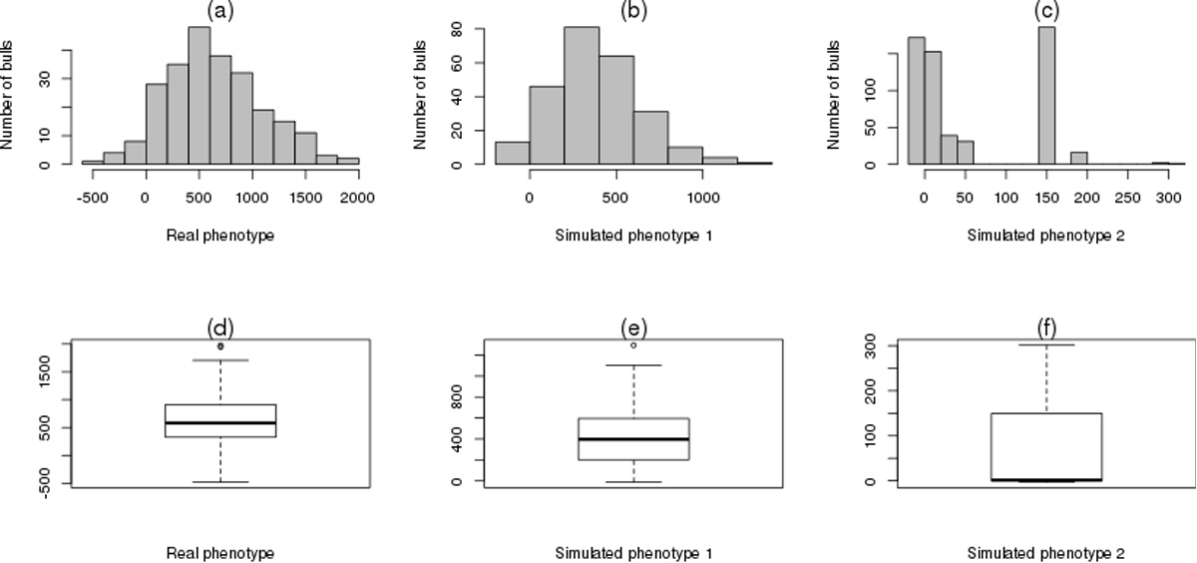
**Histogram and boxplot of the real and simulated phenotypes**. Histogram of the real phenotype (2a), simulated phenotype 1 (2b), simulated phenotype 2 (2c) and boxplots of the real phenotype (2d), simulated phenotype 1 (2e), (f) simulated phenotype 2 (2f).

In the histogram of Figure 2a, it was demonstrated that the distribution of the PTA for milk has positive skewness. From Figure 2b, it is also noticed that there are two aberrant points higher than the average of the distribution of PTA. Thereby, to verify the normality of the phenotype, we applied the normality test of Shapiro-Wilk test, which showed the same p-value of 0.01991, indicating that there is evidence that the distribution of the genetic potential does not have a normal distribution.

The simulated phenotype 1 was constructed from multiple simulations of the betas of Equation (1) to introduce an asymmetry similar to milk PTA as shown in Figure 2b. The objective of this simulation was to verify the performance of the method in the presence of markers with only main effects, ie, in a controlled environment.

The simulated 2 phenotype has empirical distribution which apparently does not fit into any theoretical probabilistic model as seen in Figure 2c, in addition to presenting a high degree of asymmetry. One of the requirements for the use of techniques of classical regression is that the independent variable must have an approximately normal distribution to proper fit of the model to the data. However, the simulation 2 was performed in order to generate aberrant effects to verify the accuracy and robustness of the method in the presence of both of non-linearities and noise.

The real and simulated phenotypes 1 have outliers above the average in Figures 2d, e, but little asymmetry. The simulated phenotype 2 doesn't show any outlier, however, it demonstrates a high level of asymmetry according to Figure 2f.

### Preprocessing

For comparison of the filters used to select the most important markers were created two database: one without and one with quality control (QC). There has been no standard preprocessing in the data without QC such as call-rate, MAF and Hardy-Weinberg equilibrium (HWE). The purpose of this was not to eliminate SNPs with small effects alone, which when combined with other SNPs, are important in the description of PTA milk.

In the case of simulated databases, no QC filters were applied because the MAF has been defined in such a way to be greater than 10% and less than 40%. Since, the threshold is 5%, all SNPs generated will satisfy it.

The parameters used by the Illumina software referring to genotype calls are standard. As for the database with QC, the applied filters simultaneously were call-rate>=0.95, MAF > = 0.05 to HWE > = 0.05/56,947, being this value the Bonferroni significance and 56,947 is the quantity of SNPs in the original database. After applying the filters described above, 22,799 markers for applying the selection method established in this study remained. If the filters were applied separately, 9,158 markers would be eliminated by call rate, 25,764 by MAF and 1,091 by HWE.

In the sample without quality control, 6,192 markers showed no information due to errors in reading the chip Ilummina Bovine 56k, leaving 50,755 markers. From them, 3 markers had no allelic variation, so they were disregarded, totaling 50,752 for subsequent analysis. In the final database consisting of 50,752 SNPs, there are 265,788 missing values, thus the same number of imputations was carried out.

### First selection of markers

For the construction of most significant groups of markers, we used the Spearman correlation coefficient, because it has some advantages over the Pearson correlation coefficient, namely: does not assume that the relationship between the two variables is linear, does not significantly change the outcome in the presence of outliers and does not require that the variables have probability distributions determined a priori [19,20]. Briefly, the Spearman coefficient is the Pearson coefficient applied to the posts of the original variables. The Equation 14 shows how to calculate the coefficient of Spearman correlation (ρ) for repeating data.

(14) $$\rho =\frac{\overset{n}{\underset{i=1}{\sum }}\left(x_{i}-\bar{x}\right)\left(y_{i}-\bar{y}\right)}{\sqrt{\overset{n}{\underset{i=1}{\sum }}{\left(x_{i}-\bar{x}\right)}^{2}{\left(y_{i}-\bar{y}\right)}^{2}}}$$

Where *n *is the sample size, $x_{i}$ and $y_{i}$ are the ranks of the original variables $X_{i}$ (SNPs markers) and $Y_{i}$ (PTA milk). For the ranking of the effects of the markers, it was evaluated the Spearman correlation coefficient of each marker with the PTA and its corresponding p-value. So, it created an increasing sequence of subsets $A_{i}$ of markers where $A_{1}\subset A_{2}\subset ...\subset A_{8}$, and the index 1 refers to the p-value 10^-9^, index 2, the p-value 10^-8^, and so on, until the index 8 which is for the largest p-value 10^-2^, as shown in Table 2. Only 8 groups were adopted because for the group of p-value 10^-1 ^the performance was the same of the group with p-value 10^-2 ^and the number of markers has greatly increased. Thus, the behavior of the accuracy of the PUK increased to the p-value 10^-2 ^and remained constant for higher p-values.

**Table 2:** Number of SNPs selected from the p-value of Spearman correlation coefficient in the real database

Groups	SNP selection*	# SNPs without QC	# SNPs with QC
1	< 10^-9^	68	12
2	< 10^-8^	226	17
3	< 10^-7^	431	43
4	< 10^-6^	712	105
5	< 10^-5^	1,181	242
6	< 10^-4^	1,996	595
7	< 10^-3^	3,440	1,397
8	< 10^-2^	6,512	3,356

*SNP markers selected with a p-value less than the stipulated threshold.

For the simulated databases, we assessed 17 groups of markers, because the behavior of the performance of PUK increased to a certain point and then began to decrease, possibly due to noise generated by highly correlated markers.

## Model

The SVR regression was used to assess the explanatory power of the groups of markers in relation to the two simulated phenotypes and PTA for milk. This choice was due to its great flexibility, because the SVR does not assume linearity of the model (as long as they adopt non-linear kernel), nor normality of residuals and easily adapts to the high data dimensionality.

### Parameters

Various parameters *C *and ε of SVR models were tested with linear kernel. For the other two kernels, besides the 2 previous parameters, it was the tested specific parameter γ for the RBF kernel and the specific parameters ω and σ for the PUK. For all SVRs models, several simulations were carried out for each subset of SNPs.

### Comparison of the models

For comparison of SVR models, we used cross-validation with 10-folds on each of the 8 sets of data in Table 2. Only one Pearson correlation coefficient was calculated for each fold and this procedure was repeated 10 times with different random seed for each partition created, totaling 10 estimates for the correlation, which increases reliability in statistical comparison of models.

### Second selection of markers

Based on group 8 of the real database that generated the highest average and lowest standard deviation of the correlation, a wrapper, based on a binary genetic algorithm (GA) with fitness given by the cross-validation MSE is applied to a second selection of markers. The entire methodology used in this second variable selection is based on [15] and [16].

As the number of combinations between 6,512 markers is extremely high, we used a GA to find the "best" markers in a skilled computational time, while not guaranteeing the uniqueness of the solution "optimal" found. Thus, the objective of the GA is to check the possibility of eliminating some markers of the best set generated from the first selection, as it is believed that the GA can better assess the interactions between markers than elimination made through filters quality control standards such as call-rate, HWE, MAF and LD.

The parameters adopted for the GA used for selecting SNPs were: probabilities of crossover and mutation equal to 0.6 and 0.033 respectively, population size and number of generations equal to 20 for real database. For the simulated database without epistasis (simulation 1), the population size was increased to 100 and the number of generations to 500, because the number of markers simulated was only 1,000, consuming less processing time. The selection technique known as elitism was used to keep the best individual of the previous generation in the next generation.

Both the wrapper SVR with GA as all models of SVR were performed in Weka software version 3.7.9 [21]. All the manipulation and coding of the data were made in the R software [22] by the packages HapEstXXR, scrime, RWekaandSNPRelate. The HapEstXXR package was used for the implementation of the call rate, MAF and HWE in the set of real data. The scrime was used for the simulations 1 and 2, while the RWeka converted data frames of R to files with extension *arff*, to be evaluated in the GUI called *Experimenter*. The SNPRelate was used to generate the graphs of the matrices of LD between markers.

## Results and discussion

Following the results of the methodology applied to both simulated and real basis bases will be presented in addition to the comparison of our results with the standard method. In addition, all files generated from the first selection for the two simulated databases (simulations 1 and 2) are in sup file 2, with and without correction Bonferrroni. For details about the files in the sup file 2, see sup file 3.

### Simulated phenotype without epistasis (simulation 1)

From Table 3 we note that the 7 markers used to construct the simulated phenotype represent raw p-values ranging from 10^-10 ^to 10^-1^, however, when applied Bonferroni correction, SNPs 20, 30 and 40 are lost because they have adjusted p-value equal to 1. But these markers showed non-significant raw p-values, so they were not excluded by the Bonferroni correction. An important observation is that since the proposed method constructs intervals of p-values (raw or adjusted), SNPs with p-values close to 1, but smaller, they can be selected. However, when the Bonferroni correction adjusts the raw p-value of the marker matching it to 1, the method will never select it. If the threshold of the p-value is equal to 0.05, only the SNP5 would be eliminated. Nevertheless, if the same threshold is considered, we would have at least 119 SNPs as can be seen in line 9 of Table 5. The possible benefit of the suggested method is initially to allow SNPs with insignificant p-values, but which have association with the phenotype, are selected for the first selection. Once there is such flexibility, a second selection is required to eliminate noisy SNPs that were introduced on the first selection.

**Table 3:** Raw and adjusted p-values of the Spearman correlation of the SNPs main in the simulated database 1.

SNP	Raw p-value	Adjusted p-value*
1	1.658460e-13	1.658460e-10
10	5.737988e-10	5.737988e-07
20	1.898224e-03	1.000000e+00
30	1.073910e-02	1.000000e+00
40	5.366227e-01	1.000000e+00
50	3.520811e-04	3.520811e-01
60	7.062554e-06	7.062554e-03

*Adjusted p-values are calculated multiplying raw p-values by total number SNPs which in this case are 1,000 markers.

**Table 5:** Mean and standard deviation of the Pearson correlation for the SVR models constructed from subsets of SNPs selected by raw p-values of the simulated database 1.

Group	SNP selection with raw p-values	# SNPs	Linear**	RBF**	PUK**
1	< 10^-9^	2^a^	0.49(0.14)	0.48(0.14)	0.54(0.14)
2	< 10^-8^	2^a^	0.49(0.14)	0.48(0.14)	0.54(0.14)
3	< 10^-7^	2^a^	0.49(0.14)	0.48(0.14)	0.54(0.14)
4	< 10^-6^	2^a^	0.49(0.14)	0.48(0.14)	0.54(0.14)
5	< 10^-5^	3^b^	0.49(0.14)	0.55(0.14)	0.56(0.13)
6	< 10^-4^	3^b^	0.49(0.14)	0.55(0.14)	0.56(0.13)
7	< 10^-3^	6^c^	0.55(0.14)	0.58(0.14)	0.71(0.16)
8	< 10^-2^	14	0.67(0.13)	0.67(0.13)	0.73(0.12)
9	< 10^-1^	119	0.49(0.14)	0.55(0.14)	0.56(0.13)
**10**	** < 0.20**	**220**	**0.36(0.18)**	**0.69(0.09)**	**0.73(0.09)**
11	< 0.30	307	0.50(0.09)	0.65(0.07)	0.68(0.06)
12	< 0.40	401	0.60(0.11)	0.69(0.09)	0.72(0.09)
13	< 0.50	511	0.63(0.10)	0.67(0.09)	0.67(0.10)
14	< 0.60	598	0.58(0.10)	0.62(0.10)	0.63(0.11)
15	< 0.70	702	0.54(0.12)	0.58(0.12)	0.57(0.12)
16	< 0.80	803	0.49(0.14)	0.50(0.14)	0.49(0.14)
17	< 0.90	914	0.39(0.16)	0.40(0.17)	0.39(0.17)
18	-	7^d^	0.64(0.13)	0.97(0.03)	0.96(0.04)

*Raw p-values of the Spearman correlation (without Bonferroni correction).

** Mean and standard deviation of the Pearson correlation in 10-fold cross validation.

(a) Only SNPs 1 and 10. (b) Only SNPs 1, 10 and 60. (c) Only SNPs 1, 10, 50, 60, 121 and 874. (d) Correct model with 7 relevant SNPs and without redundant SNPs (only SNPs 1, 10, 20, 30, 40, 50 and 60).

For each group defined after the first selection, a model SVR was constructed, evaluated for each kernel, through the Pearson correlation coefficient in 10-fold cross validation. The bold line indicates the best model from the first selection for PUK.

According to Table 4 the PUK showed the best result and the group 12 is the set with the least number of markers and higher average correlation. Thus, in this case, the GA was not applied, since the number of markers selected by the first filter is small.

**Table 4:** Mean and standard deviation of the Pearson correlation for the SVR models constructed from subsets of SNPs selected by adjusted p-values of the simulated database 1.

Group	SNP selection with adjusted p-values*	# SNPs	Linear**	RBF**	PUK**
1	< 10^-9^	1^a^	0.36(0.17)	0.37(0.17)	0.45(0.17)
2	< 10^-8^	1^a^	0.36(0.17)	0.37(0.17)	0.45(0.17)
3	< 10^-7^	1^a^	0.36(0.17)	0.37(0.17)	0.45(0.17)
4	< 10^-6^	1^a^	0.36(0.17)	0.37(0.17)	0.45(0.17)
5	< 10^-5^	1^a^	0.36(0.17)	0.37(0.17)	0.45(0.17)
6	< 10^-4^	1^a^	0.36(0.17)	0.37(0.17)	0.45(0.17)
7	< 10^-3^	1^a^	0.36(0.17)	0.37(0.17)	0.45(0.17)
8	< 10^-2^	1^a^	0.36(0.17)	0.37(0.17)	0.45(0.17)
9	< 10^-1^	3^b^	0.49(0.14)	0.55(0.14)	0.56(0.13)
10	< 0.20	3^b^	0.49(0.14)	0.55(0.14)	0.56(0.13)
11	< 0.30	3^b^	0.49(0.14)	0.55(0.14)	0.56(0.13)
**12**	**< 0.40**	**4^c^**	**0.53(0.15)**	**0.56(0.14)**	**0.78(0.16)**
13	< 0.50	4^c^	0.53(0.15)	0.56(0.14)	0.78(0.16)
14	< 0.60	4^c^	0.53(0.15)	0.56(0.14)	0.78(0.16)
15	< 0.70	5^d^	0.53(0.15)	0.56(0.14)	0.75(0.16)
16	< 0.80	6^e^	0.55(0.14)	0.58(0.14)	0.71(0.16)
17	< 0.90	6^e^	0.55(0.14)	0.58(0.14)	0.71(0.16)
18	-	7^f^	0.65(0.09)	0.65(0.09)	0.93(0.02)

*Adjusted p-values of the Spearman correlation with Bonferroni correction.

** Mean and standard deviation of the Pearson correlation in 10-fold cross validation.

(a) SNP1. (b) SNP1, 10 and 60. (c) SNP1, 10, 50 and 60. (d) SNP1, 10, 50, 60 and 874. (e) SNP1, 10, 50, 60, 121 and 874. (f) Correct model with 7 relevant SNPs and without redundant SNPs.

For each group defined after the first selection, a model SVR was constructed, evaluated for each kernel, through the Pearson correlation coefficient in 10-fold cross validation. The bold line indicates the best model from the first selection for PUK.

Although the average correlation PUK in the group 12 of Table 4 without Bonferroni correction is 0.78 and with Boferroni, 0.73, while, the first standard deviation is 0.16 and the second, 0.09.

The PUK showed the best result and the group 10 is the set with the least number of markers and higher average correlation as shown in Table 5. So, in this case, the GA was applied to the group 10 with PUK to eliminate redundant and noisy SNPs. In the correct model, the PUK had the second best result, but very close to the RBF kernel performance, which was the best performance.

The filter GA reduced approximately 10 times the number of SNPs, increasing the average correlation from 0.73 to 0.75, and maintaining the standard deviation for kernel PUK as shown in Table 6. The linear and RBF kernels showed a significant improvement in the correlation, however, it is natural that the PUK has a higher result, because the fitness of the GA is based only on the PUK kernel. This demonstrates that SNPs 1, 10, 20, 30 and 60 were captured in the dataset without Bonferroni correction, which would not be possible on the same basis with this fix applied, for the best subset of Table 4 includes only those SNPs with minor p-values: SNPs 1, 10, 50 and 60.

**Table 6:** Number of SNPs and model performance referring to the group 10 of the Table 5 before and after the application of the GA.

Filter	# SNPs	Linear**	RBF**	PUK**
Before GA	220	0.36(0.18)	0.69(0.09)	0.73(0.09)
**After GA**	21*	**0.66(0.13)**	**0.73(0.09)**	**0.75(0.09)**

*The selected markers are SNPs 1, 10, 15, 20, 30, 60, 158, 177, 269, 274, 391, 446, 516, 673, 686, 693, 717, 725, 739, 825 and 930.

** Mean and standard deviation of the Pearson correlation in 10-fold cross validation.

The bold line indicates the best model from the second selection for PUK.

The parameters used by the GA were: population size = 100, number of generations = 500, crossover probability = 0.60, mutation probability = 0.033, seed =1.

### Simulated phenotype with epistasis (simulation 2)

**Figure 3:**
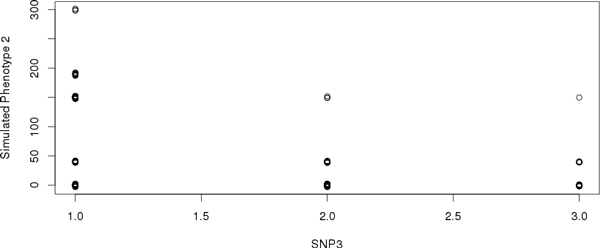
**Scatter plot between the SNP3 and simulated phenotype 2 (with epistasis)**.

Figure 3 shows that the relationship between SNP3 (the lowest p-value = 5.82e-37) and the phenotype is not linear and this is clear when the PUK and RBF kernel showed the best results in group 1 of Table 8.

**Table 8:** Mean and standard deviation of the Pearson correlation for the SVR models constructed from subsets of SNPs selected by adjusted p-values of the simulated database 2.

Group	SNP selection with adjusted p-values	# SNPs	Linear**	RBF**	PUK**
**1**	** < 10^-9^**	**1^a^**	**0.53(0.07)**	**0.94(0.04)**	**0.95(0.04)**
2	< 10^-8^	1^a^	0.53(0.07)	0.94(0.04)	0.95(0.04)
3	< 10^-7^	1^a^	0.53(0.07)	0.94(0.04)	0.95(0.04)
4	< 10^-6^	1^a^	0.53(0.07)	0.94(0.04)	0.95(0.04)
5	< 10^-5^	1^a^	0.53(0.07)	0.94(0.04)	0.95(0.04)
6	< 10^-4^	1^a^	0.53(0.07)	0.94(0.04)	0.95(0.04)
7	< 10^-3^	1^a^	0.53(0.07)	0.94(0.04)	0.95(0.04)
8	< 10^-2^	1^a^	0.53(0.07)	0.94(0.04)	0.95(0.04)
9	< 10^-1^	3^b^	0.53(0.07)	0.94(0.04)	0.95(0.04)
10	< 0.20	4^c^	0.53(0.07)	0.94(0.04)	0.95(0.04)
11	< 0.30	4^c^	0.53(0.07)	0.94(0.04)	0.95(0.04)
12	< 0.40	4^c^	0.53(0.07)	0.94(0.04)	0.95(0.04)
13	< 0.50	4^c^	0.53(0.07)	0.94(0.04)	0.95(0.04)
14	< 0.60	4^c^	0.53(0.07)	0.94(0.04)	0.95(0.04)
15	< 0.70	4^c^	0.53(0.07)	0.94(0.04)	0.95(0.04)
16	< 0.80	4^c^	0.53(0.07)	0.94(0.04)	0.95(0.04)
17	< 0.90	4^c^	0.53(0.07)	0.94(0.04)	0.95(0.04)

*Adjusted p-values of the Spearman correlation with Bonferroni correction.

** Mean and standard deviation of the Pearson correlation in 10-fold cross validation.

(a) Only the SNP3. (b) Only SNPs 3, 4 and 5. (c) Only SNPs 3, 4, 5 and 8964.

For each group defined after the first selection, a model SVR was constructed, evaluated for each kernel, through the Pearson correlation coefficient in 10-fold cross validation. The bold line indicates the best model from the first selection for PUK.

According to Table 7 SNPs 9 and 12 were eliminated from the Bonferroni correction.

**Table 7:** Raw and adjusted p-values of the Spearman correlation of the SNPs main in the simulated database 2.

SNP	Raw p-value	Adjusted p-value*
3	5.824261e-41	5.824261e-37
4	6.087710e-06	6.087710e-02
5	3.677127e-06	3.677127e-02
9	7.890460e-01	1.000000e+00
12	2.088276e-01	1.000000e+00

*Adjusted p-values are calculated multiplying raw p-values by total number SNPs which in this case are 10,000 markers.

By Tables 8 and 9, the proposed method coincides with the standard method used in GWAS, because only the SNP3 was selected due to groups with more than 1 SNP showed performance equal to or lower than group 1. Thus, it is not necessary to apply the GA as a second filter due to the existence of a single marker indicated by the first selection.

**Table 9:** Mean and standard deviation of the Pearson correlation coefficient for the simulated database 2 groups with raw p-values.

Group	SNP selection with raw p-values*	# SNPs	Linear**	RBF**	PUK**
**1**	**< 10^-9^**	**1^a^**	**0.53(0.07)**	**0.94(0.04)**	**0.95(0.04)**
2	< 10^-8^	1^a^	0.53(0.07)	0.94(0.04)	0.95(0.04)
3	< 10^-7^	1^a^	0.53(0.07)	0.94(0.04)	0.95(0.04)
4	< 10^-6^	1^a^	0.53(0.07)	0.94(0.04)	0.95(0.04)
5	< 10^-5^	3^b^	0.53(0.07)	0.94(0.04)	0.95(0.04)
6	< 10^-4^	5	0.53(0.07)	0.94(0.04)	0.94(0.04)
7	< 10^-3^	15	0.53(0.07)	0.94(0.04)	0.95(0.04)
8	< 10^-2^	99	0.64(0.07)	0.68(0.06)	0.69(0.06)
9	< 10^-1^	995	0.66(0.06)	0.79(0.04)	0.80(0.04)
10	< 0.20	2,053	0.73(0.05)	0.80(0.03)	0.80(0.03)
11	< 0.30	3,079	0.74(0.04)	0.79(0.03)	0.79(0.03)
12	< 0.40	4,066	0.75(0.04)	0.78(0.04)	0.78(0.04)
13	< 0.50	5,033	0.74(0.04)	0.75(0.04)	0.76(0.04)
14	< 0.60	5,996	0.69(0.05)	0.71(0.05)	0.71(0.04)
15	< 0.70	6,950	0.63(0.05)	0.64(0.05)	0.65(0.05)
16	< 0.80	7,934^c^	0.51(0.07)	0.51(0.07)	0.53(0.07)
17	< 0.90	8,893^c^	0.33(0.10)	0.32(0.10)	0.33(0.10)
18	-	5^d^	0.53(0.07)	0.97(0.02)	0.99(0.01)

*Raw p-values of the Spearman correlation (without Bonferroni correction).

** Mean and standard deviation of the Pearson correlation in 10-fold cross validation.

(a) Only SNP3. (b) Contains SNPs 3, 4 and 5. (c) Contains SNPs 3, 4, 5, 9 and 12. (d) Only SNPs 3, 4, 5, 9 and 12.

When we evaluated the model consisting only of 5 relevant SNPs, the average correlation of the PUK was 0.99 with standard deviation 0.01, that is, the best result among the 3 kernels reviews. According to Table 9 the class with the least amount of markers which includes all 5 SNPs is 16 with 7,934 markers, that is, this class has a lot of noisy and irrelevant information, which leads to low explanatory power in all kernels.

Therefore, if the group 16 is chosen to be analyzed, one can use the GA to reduce the number of markers and improve the performance of the PUK, aiming to converge to the average correlation of 0.99 correct model, but we did not perform this analysis because the proposed method evaluates the subset with the smallest number of SNPs with the highest average correlation.

### Real database

The first analysis was related to the accuracy of SVR models with linear kernels, radial basis function and Pearson VII Universal. From Table 10 we note that the best model with PUK kernel, both the lowest average and the lowest standard deviation of the correlation coefficient was set at p-value less than 10^-2^.

**Table 10:** Mean and standard deviation of the Pearson correlation for the SVR models constructed from subsets of SNPs selected by raw p-values of the real database without QC.

Group	SNP selection with raw p-values*	# SNPs	Linear**	RBF**	PUK**
1	< 10^-9^	68	0.60(0.14)	0.68(0.11)	0.68(0.11)
2	< 10^-8^	226	0.48(0.17)	0.72(0.09)	0.72(0.09)
3	< 10^-7^	431	0.44(0.16)	0.74(0.09)	0.75(0.08)
4	< 10^-6^	712	0.71(0.09)	0.77(0.08)	0.74(0.09)
5	< 10^-5^	1,181	0.76(0.09)	0.76(0.08)	0.78(0.08)
6	< 10^-4^	1,996	0.78(0.08)	0.74(0.08)	0.78(0.08)
7	< 10^-3^	3,440	0.80(0.08)	0.67(0.13)	0.80(0.08)
**8**	**< 10^-2^**	**6,512**	**0.81(0.08)**	**0.81(0.08)**	**0.81(0.08)**

*Raw p-values of the Spearman correlation (without Bonferroni correction).

** Standard deviation of the estimates of the 10-fold cross validation.

According to Table 11 three models SVR based on the markers of the group 8 of the database with QC showed equivalent prediction and accuracy, indicating a correlation averaging 0.80 with a standard deviation equal to 0.08. Moreover, it seems that the group 8 markers have a linear association with PTA milk because both the kernels RBF and PUK replicated this behavior.

**Table 11:** Mean and standard deviation of the Pearson correlation for the SVR models constructed from subsets of SNPs selected by raw p-values of the real database with QC.

Group	SNP selection with raw p-values*	# SNPs	Linear**	RBF**	PUK**
1	< 10^-9^	12	0.67(0.10)	0.67(0.10)	0.67(0.10)
2	< 10^-8^	17	0.64(0.10)	0.67(0.10)	0.67(0.10)
3	< 10^-7^	43	0.59(0.12)	0.68(0.09)	0.70(0.08)
4	< 10^-6^	105	0.31(0.18)	0.72(0.07)	0.71(0.07)
5	< 10^-5^	242	0.67(0.09)	0.77(0.08)	0.78(0.07)
6	< 10^-4^	595	0.77(0.08)	0.69(0.09)	0.79(0.07)
7	< 10^-3^	1,397	0.78(0.08)	0.79(0.09)	0.79(0.08)
**8**	**< 10^-2^**	**3,357**	**0.80(0.08)**	**0.75(0.08)**	**0.80(0.08)**

*Raw p-values of the Spearman correlation (without Bonferroni correction).

** Mean and standard deviation of the Pearson correlation in 10-fold cross validation.

The bold line indicates the best model from the first selection for PUK.

The Table 12 shows that the subset of markers extracted from group 8 showed higher mean correlation 0.84 with a standard deviation slightly lower 0.07 for the PUK kernel. This shows a significant gain in the use of GA for selecting the most informative SNPs without QC. However, in the database with QC, there was a small increase in the mean correlation and was kept the same standard deviation 0.08.

**Table 12:** Mean and standard deviation of the correlation coefficient of Pearson in 10-folds with 10 repetitions in the best subset found by GA with the same parameters used for the group 8 of the Table 11

Real Database	# SNPs	Kernel		
		Linear	RBF	PUK
Database without QC before GA	6,512	0.81 (0.08)	0.81(0.08)	0.81 (0.08)

**Database without QC after GA**	**3,073**	**0.84(0.07)**	**0.67(0.14)**	**0.84(0.07)**

Database with QC before GA	3,357	0.80(0.08)	0.75(0.08)	0.80(0.08)

**Database with QC after GA**	**1,073**	**0.82(0.08)**	**0.44(0.25)**	**0.81(0.08)**

** Mean and standard deviation of the Pearson correlation in 10-fold cross validation.

The bold lines indicate the best model from the second selection for PUK.

The parameters used by the GA were: population size = 20, number of generations = 20, crossover probability = 0.60, mutation probability = 0.033, seed = 1.

**Figure 4:**
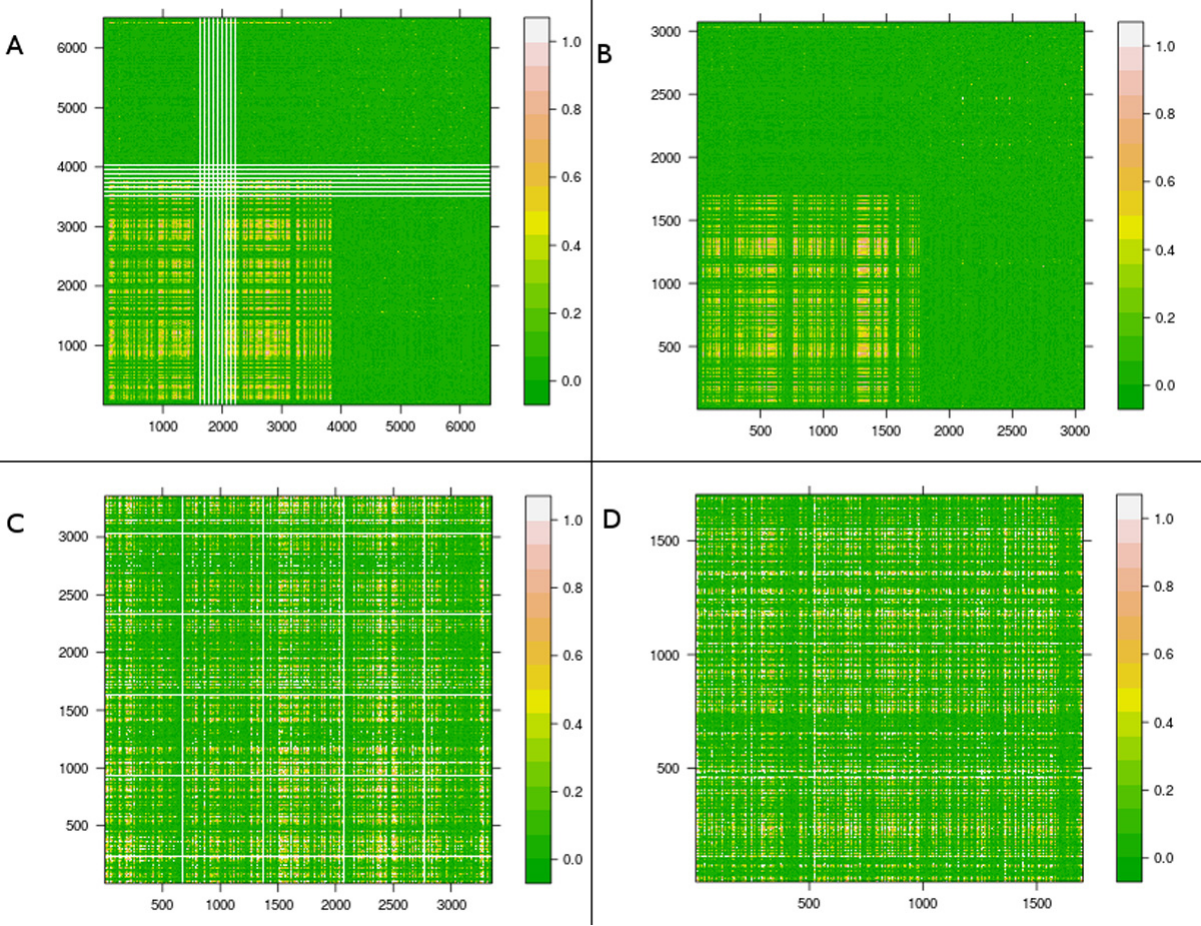
**LD matrix between markers selected by the proposed method**. LD matrix computed by r^2 ^between markers of group 8 without QC (4a) and with QC (4c), and the subsets extracted of the group 8 by GA without QC (4b) and with QC (4d). The color scale is interpreted as follows: high LD is white and low LD is green.

In Figure 4 we can see that the GA was able to eliminate several redundant markers (high LD) from group 8 of the database without QC because the white lines in Figure 4a was completely eliminated as it is observed from Figure 4b. Moreover, the yellow colored region (correlation between 0.4 and 0.6) of Figure 4a is reduced compared to Figure 4b. In relation to the database with QC, the GA also eliminated several markers highly correlated as Figures 4c and 4d. However, the final set of markers also indicates some degree of correlation. From these observations, it seems that the filters applied in the group 8 of the 2 real databases eliminated several non- informative markers correlated with the others.

In relation to the work of [2] and [6], there is a clear gain in using the PUK kernel over other kernels analyzed on them, as [6] uses the linear and RBF kernels with default values in R software, that is, the parameters SVM have not been optimized. In the case of [2], only the RBF kernel was studied, and to find the "best" parameters *C *and γ, there was an extensive search grid. However, neither of the two studies extracts from groups, constructed from the p-value, the "best" subset explanation for the phenotype and this was accomplished by applying the technique of variable selection wrapper based on the prediction error of the SVR as fitness GA.

The PUK kernel proved to be robust to capture the behavior of linear and RBF kernels, as long as the appropriate parameters are used. Therefore, on the methodology proposed in this paper, it is necessary to assess the performance of the SVR with PUK kernel. However, regardless the kernel adopted, the mathematical formulation of the SVR brings a disadvantage regarding the biological interpretation.

The method developed in this study indicated that the 68 most significant markers of group 1 without QC has low predictive power and low accuracy compared to PTA milk and the subgroup with 3,073 markers showed high prediction and greater accuracy. This may indicate that the PTA milk is a phenotype that is influenced by several markers with small effects on it, besides the possibility of epistasis and dominance, however, such genetic effects cannot be proven by the method suggested in this work.

The SVR model with kernel PUK of groups 8 with and without QC showed high predictive power even in the presence of non-normality in the dependent variable PTA for milk, and have similar performance, and accuracy. However, when applied the filter of the GA, the best subset generated from group 8 without QC was higher both in prediction and accuracy as compared to group 8 with QC. This fact seems to show that the group 8 without QC has sufficient and necessary for the explanation of the phenotype and the group 8 with QC has markers necessary, but not sufficient.

## Conclusions and future works

The method developed in this work demonstrated robustness, because the initial set of markers without QC was composed of approximately 50,752 markers and reached up to 3,073 at the end of the selection process, ensuring good accuracy and high accuracy for the SVR model with PUK kernel. At the base without QC, the method found 1,073 markers in total 22,799 SNPs, maintaining the same average correlation. This fact may indicate a trait with polygenic effect as in [23]. In addition to this fact, the GA was able to eliminate most of the redundancy in the databases with and without QC. However, the remaining issue is to understand what level of redundancy that should remain the LD between markers and this can be exploited by analyzing other thresholds or can be modeled by means of linguistic variables of the fuzzy logic in future work.

In the simulated database 1, the suggested method found 21 SNPs, 5 belonging to the group of 7 most important, while the standard method indicated only 1. Thus, the polygenic effect was mapped very accurately, however, to find only 7 markers, it is necessary to perform a sensitivity analysis on the parameters of the GA (population size, number of generations, probabilities of crossover and mutation) in an attempt to eliminate the noise of the 16 false-positive SNPs.

In the simulated database 2, the standard and proposal methodologies indicated only SNP3 as relevant marker, where 5 SNPs is the correct number. Thus, we have only chance to find the top 5 SNPs, analyzing the subgroup 16 with 7,934 SNPs, however, this group showed one of the lowest correlations. This indicates that the proposed method needs to be improved in scenarios with epistasis. But, the first selection by the p-value was not satisfactory, suggesting future work for the adoption of GA directly in the database without any filter, but with the introduction of LD, physical distance between markers, p-value, HWE, MAF, call-rate in the fitness of the GA in order to improve the convergence of GA to the "optimal" subset of markers.

Another point to be analyzed in depth is SNPs that were not eliminated in the database without QC and can bring a high level of noise through the imputation used, inflating the explanatory power of SVR in 8 group.

The standard filters (call rate, MAF and HWE) used in the database with QC seem to delete markers essential to explaining the phenotype PTA milk. From this study it is necessary to understand which filters are responsible for eliminating the most relevant SNPs. Therefore, the results obtained are promising for the application in GWAS, because most of the works in this area apply standard filters for preprocessing the database. Furthermore, the Bonferroni correction excluded essential markers in the simulated database, indicating that this "protection" for the inflation of type I error can impair association studies.

No change in the values of real and simulated phenotypes in order to make your normal distribution was necessary because the PUK showed 0.95 (last line in Table 4) and 0.99 (last line in Table 9) the average correlation in the simulated models 1 and 2 respectively. This feature is extremely important for the automation of the methodology and the specialist knowledge is unnecessary to correct processing of the data.

A potential improvement in the general settings of the PUK would be the application of the methodology suggested in [24], which uses a genetic algorithm with the simplex method proposed by Nelder-Mead to find simultaneously all the parameters needed for any kernel SVR.

There is the possibility to adapt the method for problems classification, ie, case-control studies, for example, for disease type 1 diabetes, simply, change the p-value of the Spearman correlation for the p-value of chi-square test between the SNP and the phenotype. Simply change the p-value of the Spearman correlation, for example, the p-value of chi-square test between the SNP and the phenotype. Moreover, the methodology proposed here can be adapted for genomic selection aiming at predicting the breeding value of the individual from the genotype.

Future work is necessary to determine fundamental physical map in which distances between 3,073 markers to verify that many of the markers are found indicating the same region or distinct regions in the genome, but this will be accomplished. Furthermore, to verify the efficiency of the method developed here is required application in other databases SNPs associated with different phenotypes.

As future work, the level of non-linearity of the database could be assessed by a measure based on the parameters ω and σ the PUK. Thus, it is possible to indicate whether the database has epistasis (non-linear interactions) or not based on this metric. But that will be developed later.

## Competing interests

The authors declare that they have no competing interests.

## Authors' contributions

FCO (PhD student, Graduate Program in Computational Modeling, UFJF) proposed to use the Spearman correlation instead of simple linear regression and SVR for evaluating groups of markers making the model multi-attribute; CCHB (Department of Computer Science, UFJF) is the team leader in this work, gave valuable contributions to the analysis of the method and on the possibilities for future works; FNA (National Research Center of Dairy Cattle, Embrapa - FAPEMIG, Juiz de Fora - Brasil) revised the text and contributed to the methodology of this study; FFS (Department of Animal Science, UFV) proposed to carry out the initial selection based on *p-value *ranges for the construction of SVR; RVS (National Research Center of Dairy Cattle, Embrapa) provided the real database; MVGBS (National Research Center of Dairy Cattle, Embrapa) contributed to the genomic approach and the application of the quality control filters; WA (National Research Center of Dairy Cattle, Embrapa and Department of Computer Science, UFJF) is the project leader, proposed the general approach of this study and the use of SVR and GA for the second selection markers and other computational intelligence techniques described in the future works. All authors read and approved the final manuscript.

## Supplementary Material

Sup file 1 **Simulated databases 1 and 2**. R scripts for generating the genotype and phenotype from a linear model with only additive effects (simulation 1) and additive effects with epistasis (simulation 2). Furthermore, it was made the first selection of markers in data frames for later conversion into weka ARFF format used in the second selection by GA with SVR in Weka. Format: RAR Size: 3,45 KB Click here for file

Sup file 2 **Databases after the first selection of markers first selection in the simulations 1 and 2**. Format: RAR Size: 6,92 MB Click here for file

Sup file 3 **Additional explications of the files contained in the sup file 2**. Format: DOC Size: 51,5 KB Click here for file

## Declarations

Publication costs for this work were funded by Federal University of Juiz de Fora (UFJF).

This article has been published as part of *BMC Genomics *Volume 15 Supplement 7, 2014: Proceedings of the 9th International Conference of the Brazilian Association for Bioinformatics and Computational Biology (X-Meeting 2013). The full contents of the supplement are available online at http://www.biomedcentral.com/bmcgenomics/supplements/15/S7.
