# Supplementary material for: SNPs selection using support vector regression and genetic algorithms in GWAS
Source: BMC Genomics. 2014 Oct 27;15(Suppl 7):S4. doi: 10.1186/1471-2164-15-S7-S4 (PMC4243330; doi:10.1186/1471-2164-15-S7-S4)
Supplement: Sup file 3 — Additional explications of the files contained in the sup file 2. Format: DOC Size: 51,5 KB [file 1471-2164-15-S7-S4-S3.doc]

### Additional file 1 – Database after the first selection of markers with p-value < 10^-9^ with Bonferroni correction for simulation 1

File: data_s_10_9_sim1.arff

Format: ARFF Size: 4 KB

### Additional file 2 – Database after the first selection of markers with p-value < 10^-8^ with Bonferroni correction for simulation 1

File: data_s_10_8_sim1.arff

Format: ARFF Size: 4 KB

### Additional file 3 – Database after the first selection of markers with p-value < 10^-6^ with Bonferroni correction for simulation 1

File: data_s_10_6_sim1.arff

Format: ARFF Size: 4 KB

### Additional file 4 – Database after the first selection of markers with p-value < 10^-5^ with Bonferroni correction for simulation 1

File: data_s_10_5_sim1.arff

Format: ARFF Size: 4 KB

### Additional file 5 – Database after the first selection of markers with p-value < 10^-4^ with Bonferroni correction for simulation 1

File: data_s_10_4_sim1.arff

Format: ARFF Size: 4 KB

### Additional file 6 – Database after the first selection of markers with p-value < 10^-3^ with Bonferroni correction for simulation 1

File: data_s_10_3_sim1.arff

Format: ARFF Size: 4 KB

### Additional file 7 – Database after the first selection of markers with p-value < 10^-2^ with Bonferroni correction for simulation 1

File: data_s_10_2_sim1.arff

Format: ARFF Size: 5 KB

### Additional file 8 – Database after the first selection of markers with p-value < 10^-1^ with Bonferroni correction for simulation 1

File: data_s_10_1_sim1.arff

Format: ARFF Size: 5 KB

### Additional file 9 – Database after the first selection of markers with p-value < 0.20 with Bonferroni correction for simulation 1

File: data_s_0.20_sim1.arff

Format: ARFF Size: 6 KB

### Additional file 10 – Database after the first selection of markers with p-value < 0.30 with Bonferroni correction for simulation 1

File: data_s_0.30_sim1.arff

Format: ARFF Size: 6 KB

### Additional file 11 – Database after the first selection of markers with p-value < 0.40 with Bonferroni correction for simulation 1

File: data_s_0.40_sim1.arff

Format: ARFF Size: 6 KB

### Additional file 12 – Database after the first selection of markers with p-value < 0.50 with Bonferroni correction for simulation 1

File: data_s_0.50_sim1.arff

Format: ARFF Size: 6 KB

### Additional file 13 – Database after the first selection of markers with p-value < 0.60 with Bonferroni correction for simulation 1

File: data_s_0.60_sim1.arff

Format: ARFF Size: 6 KB

### Additional file 14 – Database after the first selection of markers with p-value < 0.70 with Bonferroni correction for simulation 1

File: data_s_0.70_sim1.arff

Format: ARFF Size: 6 KB

### Additional file 15 – Database after the first selection of markers with p-value < 0.80 with Bonferroni correction for simulation 1

File: data_s_0.80_sim1.arff

Format: ARFF Size: 5 KB

### Additional file 16 – Database after the first selection of markers with p-value < 0.90 with Bonferroni correction for simulation 1

File: data_s_0.90_sim1.arff

Format: ARFF Size: 5 KB

### Additional file 17 – Database after the first selection of markers with p-value < 10^-9^ without Bonferroni correction for simulation 1

File: data_s_10_9_sim1_without_Bonferroni.arff

Format: ARFF Size: 4 KB

### Additional file 18 – Database after the first selection of markers with p-value < 10^-8^ without Bonferroni correction for simulation 1

File: data_s_10_8_sim1_without _Bonferroni.arff

Format: ARFF Size: 4 KB

### Additional file 19 – Database after the first selection of markers with p-value < 10^-7^ without Bonferroni correction for simulation 1

File: data_s_10_7_sim1_without _Bonferroni.arff

Format: ARFF Size: 4 KB

### Additional file 20 – Database after the first selection of markers with p-value < 10^-6^ without Bonferroni correction for simulation 1

File: data_s_10_6_sim1_without _Bonferroni.arff

Format: ARFF Size: 4 KB

### Additional file 21 – Database after the first selection of markers with p-value < 10^-5^ without Bonferroni correction for simulation 1

File: data_s_10_5_sim1_without _Bonferroni.arff

Format: ARFF Size: 5 KB

### Additional file 22 – Database after the first selection of markers with p-value < 10^-4^ without Bonferroni correction for simulation 1

File: data_s_10_4_sim1_without _Bonferroni.arff

Format: ARFF Size: 5 KB

### Additional file 23 – Database after the first selection of markers with p-value < 10^-3^ without Bonferroni correction for simulation 1

File: data_s_10_3_sim1_without _Bonferroni.arff

Format: ARFF Size: 6 KB

### Additional file 24 – Database after the first selection of markers with p-value < 10^-2^ without Bonferroni correction for simulation 1

File: data_s_10_2_sim1_without _Bonferroni.arff

Format: ARFF Size: 10 KB

### Additional file 25 – Database after the first selection of markers with p-value < 10^-1^ without Bonferroni correction for simulation 1

File: data_s_10_1_sim1_without _Bonferroni.arff

Format: ARFF Size: 64 KB

### Additional file 26 – Database after the first selection of markers with p-value < 0.20 without Bonferroni correction for simulation 1

File: data_s_0.20_sim1_without _Bonferroni.arff

Format: ARFF Size: 116 KB

### Additional file 27 – Database after the first selection of markers with p-value < 0.30 without Bonferroni correction for simulation 1

File: data_s_0.30_sim1_without _Bonferroni.arff

Format: ARFF Size: 374 KB

### Additional file 28 – Database after the first selection of markers with p-value < 0.40 without Bonferroni correction for simulation 1

File: data_s_0.40_sim1_without _Bonferroni.arff

Format: ARFF Size: 209 KB

### Additional file 29 – Database after the first selection of markers with p-value < 0.50 without Bonferroni correction for simulation 1

File: data_s_0.50_sim1_without _Bonferroni.arff

Format: ARFF Size: 266 KB

### Additional file 30 – Database after the first selection of markers with p-value < 0.60 without Bonferroni correction for simulation 1

File: data_s_0.60_sim1_without _Bonferroni.arff

Format: ARFF Size: 310 KB

### Additional file 31 – Database after the first selection of markers with p-value < 0.70 without Bonferroni correction for simulation 1

File: data_s_0.70_sim1_without _Bonferroni.arff

Format: ARFF Size: 364 KB

### Additional file 32 – Database after the first selection of markers with p-value < 0.80 without Bonferroni correction for simulation 1

File: data_s_0.80_sim1_without _Bonferroni.arff

Format: ARFF Size: 416 KB

### Additional file 33 – Database after the first selection of markers with p-value < 0.90 without Bonferroni correction for simulation 1

File: data_s_0.90_sim1_without _Bonferroni.arff

Format: ARFF Size: 473 KB

### Additional file 34 – Correct model with only 7 markers of simulation 1

File: correct_model_sim1.arff

Format: ARFF Size: 14 KB

### Additional file 35 – Database after the first selection of markers with p-value < 10^-9^ with Bonferroni correction for simulation 2

File: data_s_10_9_sim2.arff

Format: ARFF Size: 8 KB

### Additional file 36 – Database after the first selection of markers with p-value < 10^-8^ with Bonferroni correction for simulation 2

File: data_s_10_8_sim2.arff

Format: ARFF Size: 8 KB

### Additional file 37 – Database after the first selection of markers with p-value < 10^-7^ with Bonferroni correction for simulation 2

File: data_s_10_7_sim2.arff

Format: ARFF Size: 8 KB

### Additional file 38 – Database after the first selection of markers with p-value < 10^-6^ with Bonferroni correction for simulation 2

File: data_s_10_6_sim2.arff

Format: ARFF Size: 8 KB

### Additional file 39 – Database after the first selection of markers with p-value < 10^-5^ with Bonferroni correction for simulation 2

File: data_s_10_5_sim2.arff

Format: ARFF Size: 8 KB

### Additional file 40 – Database after the first selection of markers with p-value < 10^-4^ with Bonferroni correction for simulation 2

File: data_s_10_4_sim2.arff

Format: ARFF Size: 8 KB

### Additional file 41 – Database after the first selection of markers with p-value < 10^-3^ with Bonferroni correction for simulation 2

File: data_s_10_3_sim2.arff

Format: ARFF Size: 8 KB

### Additional file 42 – Database after the first selection of markers with p-value < 10^-2^ with Bonferroni correction for simulation 2

File: data_s_10_2_sim2.arff

Format: ARFF Size: 8 KB

### Additional file 43 – Database after the first selection of markers with p-value < 10^-1^ with Bonferroni correction for simulation 2

File: data_s_10_1_sim2.arff

Format: ARFF Size: 10 KB

### Additional file 44 – Database after the first selection of markers with p-value < 0.20 with Bonferroni correction for simulation 2

File: data_s_0.20_sim2.arff

Format: ARFF Size: 11 KB

### Additional file 45 – Database after the first selection of markers with p-value < 0.30 with Bonferroni correction for simulation 2

File: data_s_0.30_sim2.arff

Format: ARFF Size: 11 KB

### Additional file 46 – Database after the first selection of markers with p-value < 0.40 with Bonferroni correction for simulation 2

File: data_s_0.40_sim2.arff

Format: ARFF Size: 12 KB

### Additional file 47 – Database after the first selection of markers with p-value < 0.50 with Bonferroni correction for simulation 2

File: data_s_0.50_sim2.arff

Format: ARFF Size: 12 KB

### Additional file 48– Database after the first selection of markers with p-value < 0.60 with Bonferroni correction for simulation 2

File: data_s_0.60_sim2.arff

Format: ARFF Size: 12 KB

### Additional file 49 – Database after the first selection of markers with p-value < 0.70 with Bonferroni correction for simulation 2

File: data_s_0.70_sim2.arff

Format: ARFF Size: 12 KB

### Additional file 50 – Database after the first selection of markers with p-value < 0.80 with Bonferroni correction for simulation 2

File: data_s_0.80_sim2.arff

Format: ARFF Size: 12 KB

### Additional file 51 – Database after the first selection of markers with p-value < 0.90 with Bonferroni correction for simulation 2

File: data_s_0.90_sim2.arff

Format: ARFF Size: 12 KB

### Additional file 52 – Database after the first selection of markers with p-value < 10^-9^ without Bonferroni correction for simulation 2

File: data_s_10_9_sim2_without_Bonferroni.arff

Format: ARFF Size: 8 KB

### Additional file 53 – Database after the first selection of markers with p-value < 10^-6^ without Bonferroni correction for simulation 2

File: data_s_10_6_sim2_without_Bonferroni.arff (The databases with p-value < 10^-8^ and p-value < 10^-7^ showed no selected markers.)

Format: ARFF Size: 8 KB

### Additional file 54 – Database after the first selection of markers with p-value < 10^-5^ without Bonferroni correction for simulation 2

File: data_s_10_5_sim2_without_Bonferroni.arff

Format: ARFF Size: 10 KB

### Additional file 55 – Database after the first selection of markers with p-value < 10^-4^ without Bonferroni correction for simulation 2

File: data_s_10_4_sim2_without_Bonferroni.arff

Format: ARFF Size: 12 KB

### Additional file 56 – Database after the first selection of markers with p-value < 10^-3^ without Bonferroni correction for simulation 2

File: data_s_10_3_sim2_without_Bonferroni.arff

Format: ARFF Size: 24 KB

### Additional file 57 – Database after the first selection of markers with p-value < 10^-2^ without Bonferroni correction for simulation 2

File: data_s_10_2_sim2_without_Bonferroni.arff

Format: ARFF Size: 125 KB

### Additional file 58 – Database after the first selection of markers with p-value < 10^-1^ without Bonferroni correction for simulation 2

File: data_s_10_1_sim2_without_Bonferroni.arff

Format: ARFF Size: 1,199 KB

### Additional file 59 – Database after the first selection of markers with p-value < 0.20 without Bonferroni correction for simulation 2

File: data_s_0.20_sim2_without_Bonferroni.arff

Format: ARFF Size: 2,466 KB

### Additional file 60 – Database after the first selection of markers with p-value < 0.30 without Bonferroni correction for simulation 2

File: data_s_0.30_sim2_without_Bonferroni.arff

Format: ARFF Size: 3,695 KB

### Additional file 61 – Database after the first selection of markers with p-value < 0.40 without Bonferroni correction for simulation 2

File: data_s_0.40_sim2_without_Bonferroni.arff

Format: ARFF Size: 4,878 KB

### Additional file 62 – Database after the first selection of markers with p-value < 0.50 without Bonferroni correction for simulation 2

File: data_s_0.50_sim2_without_Bonferroni.arff

Format: ARFF Size: 6,037 KB

### Additional file 63 – Database after the first selection of markers with p-value < 0.60 without Bonferroni correction for simulation 2

File: data_s_0.60_sim2_without_Bonferroni.arff

Format: ARFF Size: 7,190 KB

### Additional file 64 – Database after the first selection of markers with p-value < 0.70 without Bonferroni correction for simulation 2

File: data_s_0.70_sim2_without_Bonferroni.arff

Format: ARFF Size: 8,333 KB

### Additional file 65 – Database after the first selection of markers with p-value < 0.80 without Bonferroni correction for simulation 2

File: data_s_0.80_sim2_without_Bonferroni.arff

Format: ARFF Size: 9,512 KB

### Additional file 66 – Database after the first selection of markers with p-value < 0.90 without Bonferroni correction for simulation 2

File: data_s_0.90_sim2_without_Bonferroni.arff

Format: ARFF Size: 10,781 KB

### Additional file 67 – Correct model with only 5 markers of simulation 2

File: correct_model_sim2.arff

Format: ARFF Size: 14 KB
